# Integrating informatics tools and portable sequencing technology for rapid detection of resistance to anti-tuberculous drugs

**DOI:** 10.1186/s13073-019-0650-x

**Published:** 2019-06-24

**Authors:** Jody E. Phelan, Denise M. O’Sullivan, Diana Machado, Jorge Ramos, Yaa E. A. Oppong, Susana Campino, Justin O’Grady, Ruth McNerney, Martin L. Hibberd, Miguel Viveiros, Jim F. Huggett, Taane G. Clark

**Affiliations:** 1 Faculty of Infectious and Tropical Diseases, London School of Hygiene & Tropical Medicine, London, WC1E 7HT UK; 2 Molecular Biology, LGC Ltd, Queens Road, Teddington, Middlesex, TW11 0LY UK; 3 Global Health and Tropical Medicine, GHTM, Instituto de Higiene e Medicina Tropical, IHMT, Universidade NOVA de Lisboa, UNL, Lisbon, Portugal; 4 Norwich Medical School, University of East Anglia, Norwich Research Park, Norwich, NR4 7TJ UK; 5 Department of Medicine, University of Cape Town, Observatory, Cape Town, 7925 South Africa; 6 School of Biosciences & Medicine, Faculty of Health & Medical Science, University of Surrey, Guildford, GU2 7XH UK; 7 Faculty of Epidemiology and Population Health, London School of Hygiene & Tropical Medicine, London, WC1E 7HT UK

**Keywords:** Drug resistance, Tuberculosis, Diagnostics, Drug-susceptibility testing, MDR-TB, XDR-TB, WGS

## Abstract

**Background:**

*Mycobacterium tuberculosis* resistance to anti-tuberculosis drugs is a major threat to global public health. Whole genome sequencing (WGS) is rapidly gaining traction as a diagnostic tool for clinical tuberculosis settings. To support this informatically, previous work led to the development of the widely used *TBProfiler* webtool, which predicts resistance to 14 drugs from WGS data. However, for accurate and rapid high throughput of samples in clinical or epidemiological settings, there is a need for a stand-alone tool and the ability to analyse data across multiple WGS platforms, including Oxford Nanopore MinION.

**Results:**

We present a new command line version of the *TBProfiler* webserver, which includes hetero-resistance calling and will facilitate the batch processing of samples. The *TBProfiler* database has been expanded to incorporate 178 new markers across 16 anti-tuberculosis drugs. The predictive performance of the mutation library has been assessed using > 17,000 clinical isolates with WGS and laboratory-based drug susceptibility testing (DST) data. An integrated MinION analysis pipeline was assessed by performing WGS on 34 replicates across 3 multi-drug resistant isolates with known resistance mutations. *TBProfiler* accuracy varied by individual drug. Assuming DST as the gold standard, sensitivities for detecting multi-drug-resistant TB (MDR-TB) and extensively drug-resistant TB (XDR-TB) were 94% (95%CI 93–95%) and 83% (95%CI 79–87%) with specificities of 98% (95%CI 98–99%) and 96% (95%CI 95–97%) respectively. Using MinION data, only one resistance mutation was missed by *TBProfiler*, involving an insertion in the *tlyA* gene coding for capreomycin resistance. When compared to alternative platforms (e.g. *Mykrobe predictor TB*, the CRyPTIC library), *TBProfiler* demonstrated superior predictive performance across first- and second-line drugs.

**Conclusions:**

The new version of *TBProfiler* can rapidly and accurately predict anti-TB drug resistance profiles across large numbers of samples with WGS data. The computing architecture allows for the ability to modify the core bioinformatic pipelines and outputs, including the analysis of WGS data sourced from portable technologies. *TBProfiler* has the potential to be integrated into the point of care and WGS diagnostic environments, including in resource-poor settings.

**Electronic supplementary material:**

The online version of this article (10.1186/s13073-019-0650-x) contains supplementary material, which is available to authorized users.

## Background

Tuberculosis disease (TB), caused by *Mycobacterium tuberculosis*, is the world’s major cause of death from an infectious agent [1]. The emergence of multi-drug-resistant tuberculosis (MDR-TB) is leading to difficulties in disease control. MDR-TB is resistance to at least rifampicin and isoniazid, and extensive drug resistance (XDR-TB) is the additional resistance to the fluoroquinolones and injectable drugs (amikacin, kanamycin and capreomycin) used to treat MDR-TB. Phenotypic methods of determining susceptibility to anti-tuberculosis drugs (DSTs) can take weeks and require culturing of *M. tuberculosis*. Drug resistance in *M. tuberculosis* is almost exclusively due to mutations (including single nucleotide polymorphisms (SNPs), insertions and deletions (indels)) in genes coding for drug targets or converting enzymes. Putative compensatory mechanisms have been described to overcome fitness impairment that arises during the accumulation of resistance-conferring mutations [2].

Molecular characterisation of resistance from the *M. tuberculosis* circular genome (size 4.4 Mb) offers a rapid alternative to traditional culture-based methods. Commercial PCR-based tests and line probe assays are available for a limited number of drugs but, with the exception of rifampicin, they have low sensitivity for detecting all possible molecular targets for resistance [3]. Due to the multiplicity of drugs used in the treatment of TB, determining the full resistance profile for a patient suspected of having drug-resistant disease requires the analysis of many genetic loci. Further, new mutations are being uncovered using genome-wide association and convergent evolution studies and revealing an important role for indels and copy number variants in drug resistance [4]. Whole genome sequencing (WGS) offers an attractive option as it simultaneously examines all loci and provides information regarding both small and large changes in the genome [3], allowing for the prediction of resistance and potentially susceptibility [5]. Third-generation portable sequencing technologies, such as Oxford Nanopore MinION [6], offer opportunities to roll out WGS as a diagnostic in the less well-resourced settings found in countries where TB is endemic. However, this requires efficient and automated informatic platforms to enable the data to be analysed without necessarily needing a trained genomics expert. For acceptance as a diagnostic tool to guide treatment of drug-resistant TB, the sequencing platforms and analytical tools employed must be robust and reliable.

Previously we released the *TBProfiler* webserver that allowed researchers to upload raw sequence data to retrieve a report with information on lineage and resistance across 14 anti-TB drugs. To date, this tool has been used to profile tens of thousands of isolates to produce high-quality reports and has been shown to outperform other software [7] and established diagnostic tools [3]. The underlying mutation library consists of 1193 polymorphisms across 32 targets conferring resistance to the 14 anti-tuberculous drugs. As our understanding of the molecular mechanisms of resistance is improving, such libraries of mutations need to be regularly updated. Further, there is a need to characterise genomic hetero-resistance in candidate loci, where both sensitive and resistance alleles of the same mutation are present in a sample. It has been shown that identifying hetero-resistance can lead to better predictions of the drug resistance phenotypes (e.g. XDR-TB [8]). More generally, whilst the web interface greatly simplifies the process of analysing raw sequence data, it may not be convenient for all settings. For example, a stand-alone tool may be useful in areas where internet access is slow or not available, or parallel profiling of hundreds of strains is required.

In this study, we update the *TBProfiler* library to include mutations for two further drugs used in the treatment of drug-resistant TB, cycloserine and delamanid. To improve the tool’s utility, a command line implementation has been developed, with hetero-resistance characterisation, and the capacity for processing of large-scale data, potentially from multiple WGS platforms (e.g. Illumina, MinION). The performance of the *TBProfiler* pipeline is compared to DST outcomes across > 17k *M. tuberculosis* strains from over 50 countries with Illumina WGS data, as well as on a subset that has undergone cutting edge MinION WGS.

## Implementation

### Resistance mutation library

New mutations were added to an existing robust *TBProfiler* library [3], with inclusion based on evidence from recent publications [4, 9, 10]. In total, 178 new mutations were added to the library across 16 drugs, including for cycloserine and delamanid, not present in the previous version of the library. This library is hosted on GitHub (https://github.com/jodyphelan/tbdb), and details on variants included can also be found in supplementary materials (Additional file 1: Data S1). GitHub hosting allows for changes in the mutation library to be discussed, tracked and visualised. Different versions of the library can be maintained using *Forks*, allowing users to experiment with the library without affecting the main project. These changes can then be merged into the main repository after the changes are reviewed. Multiple users/developers can contribute towards the library.

### In silico profiling of *M. tuberculosis* resistance phenotypes

A new *TBProfiler* tool for in silico prediction of drug resistance and strain lineage linked to the mutation library was developed using the Python computing language and well-established bioinformatic tools such as *trimmomatic*, *BWA/bowtie2* and S*AMtools*. The new pipeline can be customised (Additional file 2: Figure S1), but in its default mode, reads are trimmed using *trimmomatic (*parameters: LEADING:3 TRAILING:3 SLIDINGWINDOW:4:20 MINLEN:36) then mapped to the H37Rv reference (AL123456) using *bowtie2* (parameters: default). Variants are called using *BCFtools mpileup* (parameters: -ABq0 -Q0 -a DP, AD) and *BCFtools call* (parameters: -mg 10) and annotated using *BCFtools csq* (parameters: -p m) and is parallelised with GNU parallel [11]. Variants are annotated with *BCFtools* csq, which handles multiple variants in the same codon jointly. Annotated variants are compared to the *TBProfiler* library database. The *TBProfiler* pipeline calculates the proportion of the reads supporting each allele and reports this information, which can serve as a proxy for phenotypic hetero-resistance. Deletion calling is performed using *Delly* software [12]. The *TBProfiler* pipeline is available on GitHub (from https://github.com/jodyphelan/TBProfiler) and is easily installed through the *bioconda* channel [13]. A full set of new features can be found in supplementary materials (see Additional file 2: Table S1). *TBProfiler* report outputs are written in *json*, *txt* and *pdf* formats, with options to collate data into multi-sample reports (Additional file 2: Figure S3). The collated data can be graphically viewed on top of a phylogenetic tree using *iTOL*. Config files can be generated and uploaded to *iTOL* to visualise drug resistance types, lineage and individual drug resistance predictions.

### Sequencing data

A database of 17,239 strains for which DST and Illumina WGS raw data is published and publicly available was collated (see Additional file 2: Table S2-S4; Figure S2). In addition, *M. tuberculosis* isolates from three patients (por5–7; 11–12 replicates each) with known drug-resistant *M. tuberculosis* were cultured and DNA was extracted for Oxford Nanopore MinION sequencing. Sequencing libraries of the isolates were prepared from DNA extracts using the SQK-LWB001 Kit (Oxford Nanopore Technologies, Oxford). Briefly, 100 ng of DNA from each isolate was sheared at 6000 rpm in a g-tube (Covaris, Woburn, MA). The fragmented DNA was end-repaired and dA-tailed using NEBNext® Ultra™ II End Repair/dA-Tailing Module (New England BioLabs, Ipswich, MA) following the manufacturer’s protocol. End-prepped DNA was purified using AM-Pure XP beads (Beckman Coulter, Brea, CA) at 0.4× concentration, washed twice with 70% ethanol and eluted in nuclease-free water. Purified end-prepped DNA was incubated with Barcode Adaptor (BCA) from the SQK-LWB001 kit and NEB Blunt/TA Ligase Master Mix (New England BioLabs, Ipswich, MA) for 20 min at room temperature. The BCA-ligated DNA was once again purified using AMPure XP beads at 0.4× concentration, washed twice with 70% ethanol and eluted in nuclease-free water. Ten nanogrammes of DNA from each prep was amplified using a unique set of barcode primers provided with the SQK-LWB001 kit. The PCR conditions are summarised in the supplementary materials (see Additional file 2: Table S5). The PCR products were separately purified using AMPure XP beads at 0.4× concentration, washed twice with 70% ethanol and eluted in 10 μl of 10 mM Tris-HCl pH 8.0 with 50 mM NaCl. The barcoded libraries were pooled together to a total of 200 fmol in an equimolar ratio in 10 μl of 10 mM Tris-HCl pH 8.0 with 50 mM NaCl. The pooled library was incubated with 1 μl of RPD adapter (provided in the SQK-LWB001 kit) and incubated for 5 min at room temperature. The libraries were then loaded onto FLO-MIN106 (R9.4) flow cells following standard ONT protocols. Base calling was performed using Oxford Nanopore’s *Albacore* software using default parameters. The strains have previously been characterised both phenotypically using DST and genotypically using Illumina MiSeq and Sanger sequencing [14].

### The performance of the TBProfiler tool

To test the performance of the library, the WGS raw data for the 17,239 strains were processed through the new *TBProfiler* pipeline. The predictions from the tool were compared to the DST data (assumed to be the gold standard) and used to calculate the sensitivity and specificity of the library. The fastQ files from the MinION sequencing were also processed by *TBProfiler* (using parameters -m minION). Similarly, the predictive ability was compared to those from an alternative tool, *Mykrobe-predictor* TB tool [8], which was implemented using its command-line version (v0.5.6-0-gbd7923a-dirty; parameters: --expected_error_rate 0.15). The predictive ability for the CRyPTIC library [5] was calculated by transforming the published mutation list to a compatible library for *TBProfiler*, which was then run with default parameters.

## Results

The existing *TBProfiler* mutation library was updated to include 178 new mutations, 4 new targets and 2 new drugs. The overall number of unique mutations in the library is 1296 (see Table 1 for a summary). The *TBProfiler* pipeline was run across the ~ 17 k strains for which DST and high quality WGS data was available. These strains represent all lineages, with the majority in lineages 1 (10.9%), 2 (21.6%), 3 (16.7%) and 4 (49.5%), and the remaining isolates belonging to lineages 5, 6, 7 and *Mycobacterium bovis* (1.2%). The majority of strains (64.2%) were pan-susceptible, while 22.3% were MDR-TB and 2.0% were XDR-TB, and the remaining 11.5% were non -MDR-TB or -XDR-TB with resistance to at least one drug (termed “drug resistant”) (Additional file 2: Table S2). Drug susceptibility phenotypes for 16 drugs were collated and vary in their degree of completeness across the dataset. The most complete DSTs were available for the first line treatments such as rifampicin (*N* = 17,040; 98.8%) and isoniazid (*N* = 16,955; 98.4%), with the lowest for the second-line treatments (e.g. cycloserine, *N* = 402, 2.3%) (Additional file 2: Table S3).

**Table 1:** Summary of mutations included in the curated whole genome drug resistance *TBProfiler* library

Drug	Locus	Gene	SNPs (No. obs*)	Indels (No. obs*)
Rifampicin	Rv0667	*rpoB*	94 (57)	25 (8)
	Rv0668	*rpoC*	8 (8)	0 (0)
Isoniazid	*Rv1483*	*fabG1*	11 (5)	0 (0)
	*Rv1484*	*inhA*	13 (6)	0 (0)
	*Rv1908c*	*katG*	226 (78)	37 (9)
	*Rv2245*	*kasA*	4 (1)	0 (0)
	*Rv2428*	*ahpC*	21 (10)	0 (0)
Ethambutol	*Rv1267c*	*embR*	20 (3)	0 (0)
	*Rv3793*	*embC*	25 (4)	0 (0)
	*Rv3794*	*embA*	9 (5)	6 (0)
	*Rv3795*	*embB*	127 (52)	1 (0)
Pyrazinamide	*Rv1630*	*rpsA*	3 (1)	0 (0)
	*Rv2043c*	*pncA*	280 (221)	87 (36)
	*Rv3601c*	*panD*	10 (3)	1 (0)
Streptomycin	*Rv0682*	*rpsL*	16 (6)	0 (0)
	*Rv3919c*	*gid*	2 (1)	26 (15)
	*rrs*	*rrs*	19 (15)	0 (0)
Ethionamide	*Rv1483*	*fabG1*	3 (3)	0 (0)
	*Rv1484*	*inhA*	3 (3)	0 (0)
	*Rv3854c*	*ethA*	33 (23)	42 (39)
	*Rv3855*	*ethR*	2 (2)	0 (0)
Amikacin	*rrs*	*rrs*	6 (5)	0 (0)
Capreomycin	*Rv1694*	*tlyA*	16 (2)	13 (2)
	*rrs*	*rrs*	4 (4)	0 (0)
Kanamycin	*Rv2416c*	*eis*	10 (8)	0 (0)
	*rrs*	*rrs*	4 (4)	0 (0)
FQ	*Rv0005*	*gyrB*	26 (20)	0 (0)
	*Rv0006*	*gyrA*	21 (17)	0 (0)
PAS	*Rv2447c*	*folC*	18 (10)	0 (0)
	*Rv2671*	*ribD*	1 (1)	0 (0)
	*Rv2754c*	*thyX*	1 (1)	0 (0)
	*Rv2764c*	*thyA*	19 (9)	5 (0)
**Cycloserine**	*Rv2780*	*ald*	0 (0)	12 (10)
	*Rv3423c*	*alr*	3 (3)	0 (0)
Linezolid	*Rv0701*	*rplC*	1 (1)	0 (0)
	*rrl*	*rrl*	2 (1)	0 (0)
Bedaquiline	*Rv0678*	*Rv0678*	5 (0)	2 (1)
Clofazimine	*Rv0678*	*Rv0678*	5 (0)	2 (1)
**Delamanid**	*Rv3261*	*fbiA*	1 (0)	0 (0)

Drugs which are new to the library are bolded; *indels* insertions and deletions, *FQ* fluoroquinolones, *PAS* para-aminosalicylic acid. *Number of mutations observed in the ~ 17 k dataset

Genotypic hetero-resistance was present in 28 of the 32 drug targets (Additional file 2: Table S6), including *Rv0678,* which reflects the observed complex nature of resistance acquisition [15]. The predictive ability of *TBProfiler* across all 16 drugs was calculated by comparing the inferred resistance calls against the reported DST result (Table 2). The sensitivity ranged from 95.9% (rifampicin) to 23.8% (para-aminosalicylic acid (PAS)). The sensitivities for first-line treatments such as rifampicin, isoniazid and ethambutol were high (> 90%), but lower for pyrazinamide (87.6%). The low sensitivity for pyrazinamide could potentially be attributed to the high number of rare variants in the *pncA* gene, where almost half (292/624) of variants were unique to single isolates. These rare variants may influence resistance levels. Additionally, to calculate the performance of our approach, we assumed phenotypic DST to be the gold standard. However, incorrect DST data could explain some false results. For example, *M. bovis* is intrinsically resistant to pyrazinamide, but 30% of isolates obtained from the public domain for this study were classed as sensitive to pyrazinamide. Ethionamide sensitivity was estimated at 89.5%, while the specificity was 67.4%. The high number of false positives for ethionamide may be influenced by the level of resistance conferred by *inhA* promoter mutations. These levels may be close to, but under the critical concentration, and the subsequent DST result will not reflect this.

**Table 2:** Accuracy of the *TBProfiler* library

Drug	Total	Susceptible	Resistant	*TB Profiler* sensitivity (%)	*TB Profiler* specificity (%)
Rifampicin	17,040	12,473	4564	95.9	98.2
Isoniazid	16,955	11,599	5295	93.7	98.1
Ethambutol	15,334	12,698	2617	92.1	91.7
Pyrazinamide	12,381	10,447	1875	87.6	96.7
Streptomycin	5366	3992	1288	78.0	96.3
Ethionamide	987	649	332	89.5	67.4
Amikacin	1480	1138	342	86.0	98.3
Capreomycin	1783	1388	393	84.7	95.9
Kanamycin	1908	1252	653	92.0	96.8
Ciprofloxacin	406	342	64	90.6	98.0
Moxifloxacin	905	798	107	86.0	91.9
Ofloxacin	2060	1543	514	90.1	96.5
Fluoroquinolones	2532	1944	539	89.1	97.1
PAS	418	373	42	23.8	96.7
Cycloserine	402	295	107	43.0	92.5
MDR-TB	16,879	11,293	4151	94.1	98.3
XDR-TB	2026	1681	343	83.4	96.4

*MDR-TB* multi-drug-resistant TB, *XDR-TB* extensively drug-resistant TB, *PAS* para-aminosalicylic acid, − could not be determined

Sensitivity to the second-line injectables ranged between 84.7% for capreomycin and 92.0% for kanamycin. The sensitivity for fluoroquinolones was high and ranged from 86.0% for moxifloxacin to 90.6% for ciprofloxacin. The variants conferring resistance to the individual drugs in the fluoroquinolone class do not differ in our library, and the differences in sensitivity are attributable to the variability in DST across the drugs. The overall sensitivity for the fluoroquinolones class reported by *TBProfiler* was 89.1%. Sensitivities for PAS (23.8%) and cycloserine (43.0%) were low, indicating difficulties either with unknown molecular mechanisms or with DST. The predictive value for assigning MDR-TB and XDR-TB to isolates was high, with sensitivities at 94.1% and 83.4% respectively. Additionally, 96.5% of pan-susceptible isolates with complete phenotypic data for the first-line drugs were correctly predicted. The specificity of the library was greater than 90% for all comparisons apart from ethionamide (Table 2). The sensitivities of *Mykrobe-Profiler TB* and the library published by the CRyPTIC consortium were lower than those from *TBProfiler*, and specificities broadly similar (Additional file 2: Table S7).

To assess the ability of *TBProfiler* to perform in silico profiling using MinION data, 34 replicates underwent WGS across one MDR-TB (por5) and two XDR-TB (por6 and por7) isolates (Table 3). The median read depth after mapping was 53-fold coverage (range: 25–141) and led to on average 96.4% of the genome being covered by at least 10 reads. Across the 34 isolates and 10 drugs, there was high concordance between drug resistance mutations inferred by *TBProfiler* from the analysis of MinION and alternative Illumina and Sanger sequencing data (328/340, 94.5%). Identical mutations were identified across each set of replicates, indicating the high reproducibility of the variant calling pipeline. The discrepancies between the MinION and Illumina data were found in por7 replicates (*n* = 12), where the Illumina data revealed a frameshift insertion (751T>TTG) in the *tlyA* gene associated with capreomycin resistance. This insertion could not be called using the MinION data, due to known issues regarding indel characterisation. Allele counts from the reads mapping to position 751 in the *tlyA* gene revealed that the resistance mutation was in a minority. *Mykrobe-predictor TB* was also assessed for its ability to correctly call variants in drug resistance candidates. Greater discrepancies were observed using this pipeline, with discordant results across six drugs (Table 3).

**Table 3:** The in-silico profiling results for isolates sequenced using MinION

Samples*	Method	RIF	INH	EMB	PYR	STR	ETH	AMK	CAP	FLQ	PAS
por5	DST	R	R	R	R	R	R	S	S	S	S
	*TBProfiler*	R	R	R	R	R	R	S	S	S	S
	Mykrobe**	R	R	R	**82%R**	R	–	S	S	S	–
por6	DST	R	R	R	R	R	R	R	R	R	S
	*TBProfiler*	R	R	R	R	R	R	R	R	R	S
	*Mykrobe*	R	R	R	R	R	–	**82%R**	**82%R**	R	–
por7	DST	R	R	R	R	R	R	R	R	R	S
	*TBProfiler*	R	R	R	R	R	R	**S**	**S**	R	S
	*Mykrobe*	R	**92%R**	**S**	R	**83%R**	–	**S**	**S**	**91%R**	–

*DST* drug susceptibility testing (*R* resistant, *S* sensitive); this table shows the percentage of replicates producing the correct result; − cannot be determined; bolded values indicate the % of replicates with DST phenotypes, where resistance mutations are not found in all replicates. Underlined values indicate where the variant is not present in software mutation library; *RIF* rifampicin, *INH* isoniazid, *EMB* ethambutol, *PYR* pyrazinamide, *STR* streptomycin, *ETH* ethionamide, *AMK* amikacin, *CAP* capreomycin, *FLQ* fluoroquinolones, *PAS* para-aminosalicylic acid. *All LAM4 strain-types or sub-lineage 4.3.4.2. **Mykrobe-Profiler TB (https://github.com/iqbal-lab/Mykrobe-predictor) implemented using its command-line version

## Discussion

Advances in WGS technology have expanded a role for genome analysis in the clinical laboratory. Determining resistance to anti-tuberculosis drugs by WGS has been demonstrated as feasible and is being implemented in some specialist centres [5] where it has been found to be a cost effective option [16]. We have previously shown the robustness of variant calling tools to detect SNPs, small indels and large deletions from WGS data [14]. As WGS is adopted more widely as a diagnostic tool, there is a need for robust and reliable software tools to process the vast amounts of data generated. Additionally, the growing application of third generation sequencing platforms, such as the Oxford Nanopore MinION, has driven the need to integrate analysis options for these technologies into profiling tools to support their use in a more automated format than available currently. To aid the implementation of WGS for detecting resistance to anti-tuberculosis drugs in current clinical use, the *TBProfiler* tool has been completely rewritten to enable the rapid processing of raw sequence data using a command line interface. Flexible and editable multi-sample reports with outputs to annotate phylogenetic trees can assist with epidemiological and clinical interpretation. Additionally, evidence of hetero-resistance is now reported based on the frequency of resistant alleles in the sequence reads. However, the absence of evidence in the sequences does not rule out phenotypic hetero-resistance due to culture methods applied for obtaining DNA for sequencing. Together with the new pipeline, we provided an updated library and report a high sensitivity and specificity for MDR-TB and XDR-TB. Additionally, the tool allows for the flexible use of different libraries such as those provided by ReSeqTB [17].

*TBProfiler* includes options to analyse data from the MinION platform, which can have a high error rate, and therefore requires different tools and parameters. The MinION technology promises expanded access to WGS, due to its portability and ability to sequence directly from sputum samples [18]. As rapid sequencing from metagenomic samples to detect *M. tuberculosis* and profile resistance becomes a reality, tools to process this data are required. We demonstrated the successful application of the *TBProfiler* MinION pipeline across 34 replicates covering 3 drug-resistant isolates, which have been also undergone Illumina and Sanger sequencing. In particular, we found a high concordance between replicates and across technologies, with the only difference being an insertion in the *tlyA* gene, which suggests that it is important to go beyond SNPs for resistance prediction. More generally, as our knowledge of resistance mechanisms grows, prediction software must allow for the flexibility and customisation of resistance databases. There is a constant need to update, re-evaluate and improve mutation libraries in response to new evidence. However, a number of published mutation libraries are no longer maintained and remain static versions of evidence at the time. To circumvent this limitation, we have hosted the library on a repository that facilitates user input.

In summary, WGS has the potential to improve the resolution and timeliness of TB diagnosis, and in combination with robust DST, can lead to new insights into drug resistance mechanisms. The upgraded *TBProfiler* tool allows for the flexible and rapid analysis of WGS data from Illumina and MinION platforms to predict drug resistance and strain type profiles with high accuracy.

## Conclusions

We have shown that online and stand-alone versions of *TBProfiler* can be used to reliably profile *M. tuberculosis* drug resistance from WGS. This pipeline can be applied to data from multiple sequencing platforms and can support informatically the application of WGS as a diagnostic for TB clinical management, either in combination with culture or ultimately directly from patient samples.

### Availability and requirements

Project name: TBProfiler

Project home page: https://github.com/jodyphelan/TBProfiler

Operating system(s): Linux, OSX

Programming language: Python

Other requirements: Conda

Licence: GPL-3.0

Any restrictions to use by non-academics: None.

## Additional files

Additional file 1: **Data S1.** Mutations in the library (CSV 51 kb)

Additional file 2: **Table S1.** New features in *TBProfiler.* **Table S2**. Distribution of drug resistance types by lineage. **Table S3**. Number of tested isolates for each drug. **Table S4**. ENA project codes of isolates used in the study. **Table S5**. Raw sequence data and mapping statistics for the three MinION sequenced isolates. **Table S6**. Number of Homozygous and Heterozygous calls per target. **Table S7**. Predictive performance of the different libraries. **Figure S1**. Schematic highlighting the main steps in the *TBProfiler* pipeline. **Figure S2**. Map showing the geographic origin and the resistance types of isolates used in this study. **Figure S3**. Example of the report output for an isolate. (PDF 1169 kb)

## Data Availability

All raw sequence MinION data is available from the EBI short read archive (accession number PRJEB29732), and the Illumina project accession numbers are presented in Additional file 2: Table S4.

## Abbreviations

DST: Drug susceptibility testing
Indels: Insertions and deletions
MDR-TB: Multi-drug-resistant TB
PAS: Para-aminosalicylic acid
SNP: Single nucleotide polymorphism
TB: Tuberculosis
WGS: Whole genome sequencing
XDR-TB: Extensively drug-resistant TB
