# Supplementary material for: Integrating informatics tools and portable sequencing technology for rapid detection of resistance to anti-tuberculous drugs
Source: Genome Med. 2019 Jun 24;11:41. doi: 10.1186/s13073-019-0650-x (PMC6591855; doi:10.1186/s13073-019-0650-x)
Supplement: Supplementary file 2 — Table S1. New features in TBProfiler. Table S2. Distribution of drug resistance types by lineage. Table S3. Number of tested isolates for each drug. Table S4. ENA project codes of isolates used in the study. Table S5. Raw sequence data and mapping statistics for the three MinION sequenced isolates. Table S6. Number of Homozygous and Heterozygous calls per target. Table S7. Predictive performance of the different libraries. Figure S1. Schematic highlighting the main steps in the TBProfiler pipeline. Figure S2. Map showing the geographic origin and the resistance types of isolates used in this study. Figure S3. Example of the report output for an isolate. (PDF 1169 kb) [file 13073_2019_650_MOESM2_ESM.pdf]

S1 Table
New features in *TBProfile*r

Feature	Information
Easy installation	Available to install through C*onda*
Flexible output	Output in *json, txt, pdf,* and *html* formats with customisation of reportable metrics
Resistance mutation proportion	Estimation of the proportion of reads supporting resistance mutations, which can be used to detect hetero-resistance
Multi-sample reports	Summarising results across multiple isolates and generation of tables and configuration files for *iTOL*
Deletion calling	Use of *Delly* software to call large deletions, which may cause resistance
Easy library updating	The latest version of the *TBProfiler* mutation library can be downloaded from GitHub and loaded with a single command
Use of different databases	Different mutation libraries can be easily loaded and used instead of the standard *TBProfiler* mutation library
Oxford Nanopore Technology (ONT) data ready	Different workflow implemented on ONT data with *minimap2* software used for mapping and optimised parameters for *bcftools* variant calling
Parallelised	Most of the computationally intensive steps can be parallelised and will scale from running on a laptop to a powerful high-performance computing cluster
BAM input	Assuming the BAM files have been generated with the H37Rv (GCA_000195955.2) reference, they can be used as input to *TBProfiler*. This will allow the user to skip the most computationally intensive step of read trimming and mapping
Easy library modification and generation	The mutation library is hosted in a separate GitHub repository allowing for its easy inspection and modification. Mutations can be specified using mutations or a range of amino acids/nucleotides. Support for frameshifts, premature stop codons and large deletions is also present

S2 Table
Distribution of drug resistance types by lineage

Lineage	Total	Susceptible	Drug-resistant	MDR-TB	XDR-TB
lineage1	1885 (10.9%)	1518	210	151	6
lineage2	3721 (21.6%)	1464	486	1612	159
lineage3	2886 (16.7%)	2209	217	432	28
lineage4	8531 (49.5%)	5775	970	1634	152
lineage5	35 (0.2%)	21	6	8	0
lineage6	37 (0.2%)	31	3	3	0
lineage7	1 (0.0%)	1	0	0	0
M. bovis	129 (0.7%)	40	86	3	0
Total	17239 (100%)	11068 (64.2%)	1981 (11.5%)	3845 (22.3%)	345 (2.0%)

MDR-TB = multi-drug-resistant TB; XDR-TB = extensively-drug-resistant TB; Drug-resistant = non-MDR-TB/XDR-TB resistance

S3 Table
Number of tested isolates for each drug

Drug	No. Tested	% tested	Susceptible	Resistant	Resistant %
Rifampicin	17040	98.8	12473	4567	26.8%
Isoniazid	16955	98.4	11599	5356	31.6%
Ethambutol	15334	88.9	12698	2636	17.2%
Pyrazinamide	12381	71.8	10447	1934	15.6%
Streptomycin	5366	31.1	3992	1374	25.6%
Ethionamide	987	5.7	649	338	34.2%
Amikacin	1480	8.6	1138	342	23.1%
Capreomycin	1783	10.3	1388	395	22.2%
Kanamycin	1908	11.1	1252	656	34.4%
Ciprofloxacin	406	2.4	342	64	15.8%
Moxifloxacin	905	5.2	798	107	11.8%
Ofloxacin	2060	11.9	1543	517	25.1%
Fluoroquinolones	2532	14.7	1944	588	23.2%
PAS	418	2.4	373	45	10.8%
Cycloserine	402	2.3	295	107	26.6%
MDR-TB	16879	97.9	11293	4190	24.8%
XDR-TB	2026	11.8	1681	345	17.0%

PAS = Para-aminosalisylic acid; MDR-TB = multi-drug-resistant TB; XDR-TB = extensively-drug-resistant TB

S4 Table
ENA project codes of isolates used in the study

Project	Total Number	Susceptible	Drug-resistant	MDR-TB	XDR-TB
cryptic_nejm_2018[1]	8427	5174	824	2429	0
PRJNA282721[2]	1892	1507	290	90	5
PRJEB2794[3]	1334	1244	82	8	0
PRJEB7056	1105	887	177	41	0
PRJEB9680[2]	1062	731	80	249	2
PRJEB10385[4]	713	102	212	304	95
PRJEB2221[2]	366	334	26	6	0
PRJNA183624[5]	333	87	42	139	65
PRJEB2358[6]	325	293	30	2	0
PRJEB7669[7]	246	0	3	232	11
PRJNA235852[8]	213	158	35	20	0
PRJEB5162[9]	199	181	16	2	0
PRJNA187550[10]	161	44	0	94	23
PRJNA200335[11]	134	25	6	46	57
PRJEB11653[12]	127	14	78	35	0
PRJEB14199[13]	124	0	34	14	76
PRJEB2777[14]	98	98	0	0	0
PRJEB7281[15]	94	37	15	41	1
PRJEB15857[16]	56	22	8	26	0
PRJEB6945[17]	51	51	0	0	0
PRJEB7727[18]	47	24	17	6	0
PRJEB2424[19]	45	3	2	40	0
PRJEB2138[20]	37	9	4	14	10
PRJNA49659[21]	28	28	0	0	0
PRJNA376471[22]	19	12	0	7	0
PRJEB6276[23]	3	3	0	0	0

MDR-TB = multi-drug-resistant TB; XDR-TB = extensively-drug-resistant TB; Drug-resistant = non-MDR-TB/XDR-TB resistance

References

1. The CRyPTIC Consortium and the 100, 000 Genomes Project. Prediction of Susceptibility to First-Line Tuberculosis Drugs by DNA Sequencing. *N. Engl. J. Med.* **379**, 1403–1415 (2018).
2. Walker, T. M. *et al.* Whole-genome sequencing for prediction of Mycobacterium tuberculosis drug susceptibility and resistance: a retrospective cohort study. *Lancet Infect. Dis.* **15**, 1193–1202 (2015).
3. Guerra-Assunção, J. A. *et al.* Recurrence due to Relapse or Reinfection With *Mycobacterium tuberculosis* : A Whole-Genome Sequencing Approach in a Large, Population-Based Cohort With a High HIV Infection Prevalence and Active Follow-up. *J. Infect. Dis.* **211**, 1154–1163 (2015).
4. Coll, F. *et al.* Genome-wide analysis of multi- and extensively drug-resistant Mycobacterium tuberculosis. *Nat. Genet.* **50**, 307–316 (2018).
5. Cohen, K. A. *et al.* Evolution of Extensively Drug-Resistant Tuberculosis over Four Decades:

Whole Genome Sequencing and Dating Analysis of Mycobacterium tuberculosis Isolates from KwaZulu-Natal. *PLOS Med.* **12**, e1001880 (2015).
6. Guerra-Assunção, J. *et al.* Large-scale whole genome sequencing of *M. tuberculosis* provides insights into transmission in a high prevalence area. *Elife* **4**, (2015).
7. Eldholm, V. *et al.* Four decades of transmission of a multidrug-resistant Mycobacterium tuberculosis outbreak strain. *Nat. Commun.* **6**, 7119 (2015).
8. Manson, A. L. *et al.* Genomic analysis of globally diverse Mycobacterium tuberculosis strains provides insights into the emergence and spread of multidrug resistance. *Nat. Genet.* (2017). doi:10.1038/ng.3767
9. Walker, T. M. *et al.* Assessment of Mycobacterium tuberculosis transmission in Oxfordshire, UK, 2007-12, with whole pathogen genome sequences: an observational study. *Lancet. Respir. Med.* **2**, 285–292 (2014).
10. Zhang, H. *et al.* Genome sequencing of 161 Mycobacterium tuberculosis isolates from China identifies genes and intergenic regions associated with drug resistance. *Nat. Genet.* **45**, 1255–1260 (2013).
11. Wollenberg, K. R. *et al.* Whole-Genome Sequencing of Mycobacterium tuberculosis Provides Insight into the Evolution and Genetic Composition of Drug-Resistant Tuberculosis in Belarus. *J. Clin. Microbiol.* **55**, 457–469 (2017).
12. Phelan, J. *et al.* Mycobacterium tuberculosis whole genome sequencing and protein structure modelling provides insights into anti-tuberculosis drug resistance. *BMC Med.* **14**, 31 (2016).
13. Dheda, K. *et al.* Outcomes, infectiousness, and transmission dynamics of patients with extensively drug-resistant tuberculosis and home-discharged patients with programmatically incurable tuberculosis: a prospective cohort study. *Lancet Respir. Med.* **5**, 269–281 (2017).
14. Bryant, J. M. *et al.* Whole-genome sequencing to establish relapse or re-infection with Mycobacterium tuberculosis: a retrospective observational study. *Lancet. Respir. Med.* **1**, 786–92 (2013).
15. Merker, M. *et al.* Evolutionary history and global spread of the Mycobacterium tuberculosis Beijing lineage. *Nat. Genet.* **47**, 242–249 (2015).
16. Senghore, M. *et al.* Whole-genome sequencing illuminates the evolution and spread of multidrug-resistant TB in Southwest Nigeria. *PLoS One* **12**, e0184510 (2017).
17. Roetzer, A. *et al.* Whole Genome Sequencing versus Traditional Genotyping for Investigation of a Mycobacterium tuberculosis Outbreak: A Longitudinal Molecular Epidemiological Study. *PLoS Med.* **10**, e1001387 (2013).
18. Feuerriegel, S. *et al.* PhyResSE: a Web Tool Delineating Mycobacterium tuberculosis Antibiotic Resistance and Lineage from Whole-Genome Sequencing Data. *J. Clin. Microbiol.* **53**, 1908–1914 (2015).
19. Clark, T. G. *et al.* Elucidating emergence and transmission of multidrug-resistant tuberculosis in treatment experienced patients by whole genome sequencing. *PLoS One* **8**, e83012 (2013).
20. Casali, N. *et al.* Microevolution of extensively drug-resistant tuberculosis in Russia. *Genome Res.* **22**, 735–745 (2012).
21. Gardy, J. L. *et al.* Whole-Genome Sequencing and Social-Network Analysis of a Tuberculosis Outbreak. *N. Engl. J. Med.* **364**, 730–739 (2011).
22. Chatterjee, A., *et al*. Whole genome sequencing of clinical strains of Mycobacterium tuberculosis from Mumbai, India: A potential tool for determining drug-resistance and strain lineage. *Tuberculosis* **107**, 63–72 (2017).
23. Kohl, T. A. *et al.* Whole-genome-based Mycobacterium tuberculosis surveillance: a standardized, portable, and expandable approach. *J. Clin. Microbiol.* **52**, 2479–86 (2014).

S5 Table

Raw sequence data and mapping statistics for the three MinION sequenced isolates

Sample [replicate]	No. reads	Median read length	% reads mapped	Median depth	% Genome covered >10-fold
por5 [01]	86767	2397.5	98.1%	36	95.4%
por5 [02]	113371	1924	97.7%	36	96.3%
por5 [03]	129161	1664	95.9%	35	95.9%
por5 [04]	223319	1292	96.3%	61	92.9%
por5 [05]	157771	963	94.5%	33	96.2%
por5 [06]	91439	2049	97.6%	30	96.1%
por5 [07]	143420	1726	96.0%	39	96.1%
por5 [08]	194814	1620	96.0%	51	96.5%
por5 [09]	248833	1569	96.0%	64	97.0%
por5 [10]	90099	1766	95.8%	25	95.0%
por5 [11]	153667	941	94.8%	33	96.2%
por6 [01]	61118	3228	99.3%	37	95.0%
por6 [02]	131045	2030	97.5%	44	95.7%
por6 [03]	296157	2633	98.5%	138	96.7%
por6 [04]	109261	1975	97.5%	35	95.7%
por6 [05]	76577	3049	99.2%	44	95.8%
por6 [06]	175930	1140	95.4%	43	93.6%
por6 [07]	201410	1811	97.2%	60	96.4%
por6 [08]	165564	1995	97.6%	54	95.9%
por6 [09]	203007	2053	98.0%	71	96.9%
por6 [10]	224948	841	93.6%	42	96.7%
por6 [11]	196627	966	94.3%	42	96.5%
por7 [01]	278337	878	93.6%	53	96.6%
por7 [02]	478768	1092	93.5%	80	97.0%
por7 [03]	617965	1054	91.6%	97	97.6%
por7 [04]	263686	1365	95.2%	58	96.9%
por7 [05]	245105	1358	96.4%	68	96.4%
por7 [06]	397219	759	93.0%	67	97.1%
por7 [07]	263092	1231	95.1%	63	95.5%
por7 [08]	883226	1068	92.1%	141	97.8%
por7 [09]	264420	1953	97.3%	84	96.8%
por7 [10]	293061	1788	96.5%	80	96.8%
por7 [11]	219224	1797	96.1%	60	96.4%
por7 [12]	175784	1886	97.2%	53	97.0%

S6 Table
Number of homozygous and heterozygous calls per resistance target across the 17k samples

Gene	Homozygous resistance calls	Heterozygous calls	Heterozygous %
rpoB	4905	296	5.7
katG	4487	161	3.5
embB	3710	269	6.8
pncA	2511	436	14.8
rpsL	2166	78	3.5
rrs	1619	896	35.6
fabG1	1368	56	3.9
gyrA	1224	296	19.5
ethA	970	144	12.9
eis	584	73	11.1
embA	379	27	6.7
gid	372	13	3.4
inhA	333	14	4.0
rpoC	231	26	10.1
ahpC	161	33	17.0
folC	139	19	12.0
ethR	138	4	2.8
alr	124	6	4.6
gyrB	96	42	30.4
thyX	85	3	3.4
ald	58	20	25.6
thyA	55	4	6.8
tlyA	39	38	49.4
panD	10	4	28.6
embC	7	2	22.2
embR	6	0	0.0
Rv0678	4	17	81.0
rrl	2	0	0.0
kasA	1	0	0.0
ribD	1	0	0.0
rplC	1	1	50.0
rpsA	0	1	100.0

S7 Table
Predictive performance across different libraries

Drug	No. tests	Susceptible	Resistant	*TB Profiler* Sensitivity	*TB Profiler* Specificity	*CRyPTIC** Sensitivity	*CRyPTIC* Specificity	*Mykrobe*** Sensitivity	*Mykrobe* Specificity
Rifampicin	17040	12473	4564	95.9%	98.2%	95.9%	97.8%	93.4%	98.4%
Isoniazid	16955	11599	5295	93.7%	98.1%	92.6%	98.4%	90.3%	98.6%
Ethambutol	15334	12698	2617	92.1%	91.7%	87.0%	92.8%	85.5%	93.1%
Pyrazinamide	12381	10447	1875	87.6%	96.7%	76.2%	97.4%	44.9%	98.6%
Streptomycin	5366	3992	1288	78.0%	96.3%	-	-	75.1%	95.6%
Ethionamide	987	649	332	89.5%	67.4%	-	-	-	-
Amikacin	1480	1138	342	86.0%	98.3%	-	-	79.0%	99.1%
Capreomycin	1783	1388	393	84.7%	95.9%	-	-	73.3%	94.4%
Kanamycin	1908	1252	653	92.0%	96.8%	-	-	83.8%	98.7%
Ciprofloxacin	406	342	64	90.6%	98.0%	-	-	-	-
Moxifloxacin	905	798	107	86.0%	91.9%	-	-	-	-
Ofloxacin	2060	1543	514	90.1%	96.5%	-	-	-	-
Fluoroquinolones	2532	1944	539	89.1%	97.1%	-	-	81.4%	97.8%
PAS	418	373	42	23.8%	96.7%	-	-	-	-
Cycloserine	402	295	107	43.0%	92.5%	-	-	-	-
MDR-TB	16879	11293	4151	94.1%	98.3%	93.4%	98.5%	89.6%	98.5%
XDR-TB	2026	1681	343	83.4%	96.4%	-	-	65.4%	97.2%

MDR-TB = multi-drug-resistant TB; XDR-TB = extensively-drug-resistant TB; PAS Para-aminosalicylic acid; FQ = Fluoroquinolones; - could not be determined;
*CRyPTIC library run using *TB Profiler*
***Mykrobe-Profiler TB* (http://www.mykrobe.com/products/predictor/) implemented using its command-line version

S1 Figure

Schematic highlighting the main steps in the *TBProfiler* pipeline

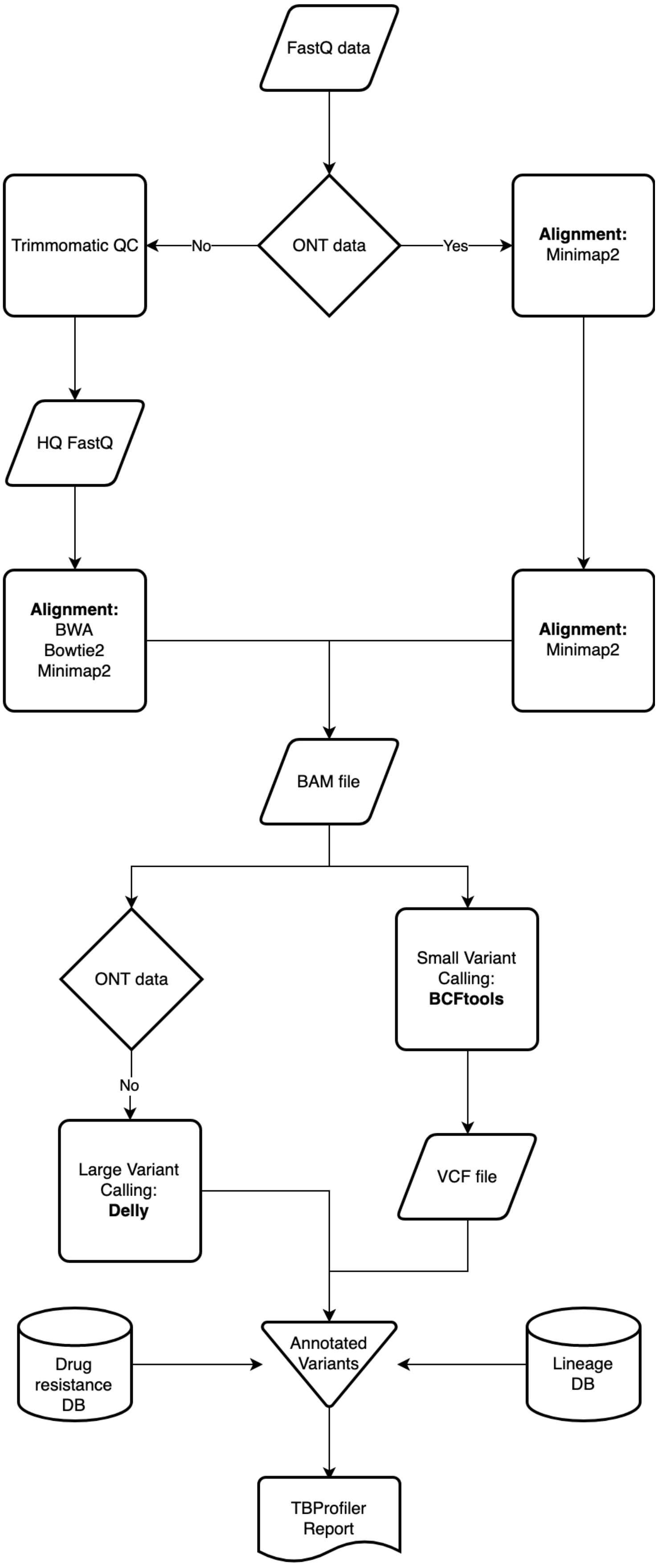

S2 Figure

Map showing the geographic origin and the resistance types of the 17k isolates used in this study

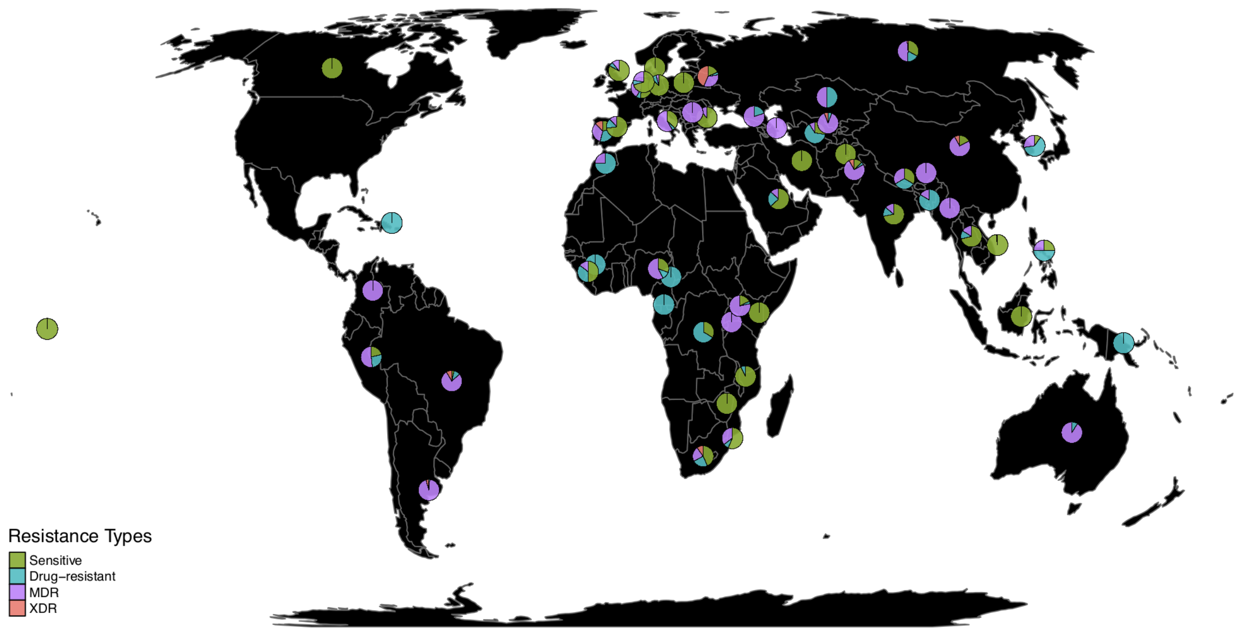

S3 Figure
Example of a report (PDF format) for an isolate

TBProfiler report

The following report has been generated by TBProfiler.

Summary

ID: tbprofiler

Date: Sat Mar 23 19:50:37 2019

Strain: lineage4.3.3

Drug-resistance: MDR

Lineage report

Lineage	Estimated Fraction	Family	Spoligotype	Rd
lineage4	1.000	Euro-American	LAM;T;S;X;H	None
lineage4.3	0.991	Euro-American (LAM)	mainly-LAM	None
lineage4.3.3	0.996	Euro-American (LAM)	LAM;T	RD115

Resistance report

Drug	Genotypic Resistance	Mutations
linezolid		
pyrazinamide	R	*pncA* Chromosome:g.2288682 2288864del (1.00)
amikacin		
levofloxacin		
clofazimine		
ciprofloxacin		
capreomycin		
streptomycin	R	*rrs* r.908a>g (0.99), *gid* Chromosome:g.4407912 4408042del (1.00)
fluoroquinolones		
isoniazid	R	*katG* p.Ser315Thr (0.98), *ahpC* r.52c>t (0.99)
ethambutol	R	*embA* r.16c>t (0.98), *embB* p.Gly406Ala (0.99)
kanamycin		
delamanid		
rifampicin	R	*rpoB* p.Ser450Leu (0.98)
ethionamide		
ofloxacin		
para-aminosalicylic acid		
bedaquiline		
moxifloxacin		
aminoglycosides		
cycloserine		

1

Other variants report

Genome Position	Locus Tag	Change	Estimated Fraction
7362	Rv0006	p.Glu21Gln	1.000
7585	Rv0006	p.Ser95Thr	1.000
8040	Rv0006	p.Gly247Ser	1.000
9304	Rv0006	p.Gly668Asp	1.000
761547	Rv0667	p.Val581Met	1.000
764995	Rv0668	c.1626C>G	0.994
781395	Rv0682	r.165t>c	1.000
1476056	rrl	r.2399g>a	0.986
1917972	Rv1694	c.33A>G	1.000
2518919	Rv2245	p.Gly269Ser	0.988
2715267	Rv2416c	c.-20 65del	0.330
3073868	Rv2764c	p.Thr202Ala	1.000
3086788	Rv2780	r.32t>c	1.000
4242643	Rv3793	c.2781C>T	1.000
4327484	Rv3855	r.65t>c	1.000
4408156	Rv3919c	p.Leu16Arg	0.977

Analysis pipeline specifications

Version: 2.0

Analysis	Program
Mapping	N/A
Variant Calling	BCFtools

Disclaimer

This tool is for **Research Use Only** and is offered free for use. The London School of Hygiene and Tropical Medicine shall have no liability for any loss or damages of any kind, however sustained relating to the use of this tool.

Citation

Coll, F. *et al.* Rapid determination of anti-tuberculosis drug resistance from whole-genome sequences. *Genome Medicine* 7, 51. 2015

2
